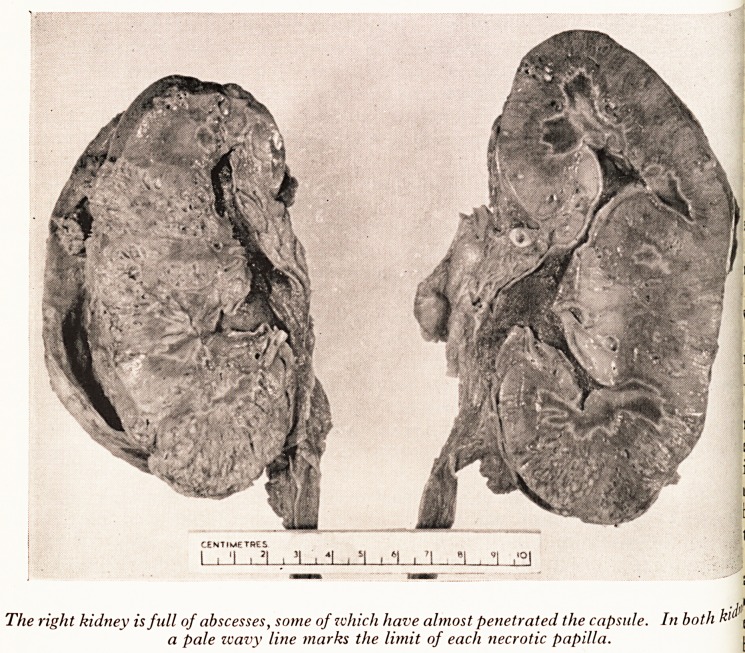# Diabetes Mellitus with Renal Papillary Necrosis

**Published:** 1957-01

**Authors:** T. F. Hewer


### DIABETES MELLITUS WITH RENAL PAPILLARY NECROSIS

*A Clinical Pathological Conference of the University of Bristol Medical Sc*

CHAIRMAN: PROFESSOR T. F. HEWER

*Professor Hewer.* In the absence of Dr. Birrell I will ask Dr. McInnes to prese clinical features of this case.

*Dr. McInnes*: This was a married woman, aged 52 years, who was first admitt May 1955 for stabilization of her diabetes mellitus. She gave a history of prof weight loss, from 13 stone to 5 stone 7lb. in 2 years. Polyuria and thirst had present for 6 months. On examination she was found to be wasted, her blood-pre was 140/90 and her haemoglobin was 15.9 g. per cent. There was no evidence o complications of diabetes apart from absence of the deep reflexes of the lower li

She was found to have a urinary infection, her urine containing albumen, pus and coliform bacilli. This was eradicated by means of sulphonamides.

Her diabetic state was treated with a free diet and protamine zinc insulin 30 un the morning. She was discharged home at the end of a month having gained a s in weight.

She was admitted during June in hypoglycaemia and the dose of insulin was red to 15 units of P.Z.I. in the mornings.

The patient was readmitted in August with further weight loss and abdominal She now only weighed 5st. 11lb. It may be said at this stage that she was an unin gent, difficult woman. She was continually forgetting to eat and her insulin had given daily by the district nurse.

On this occasion it was thought that a mass could be felt in the lower abdomen she was referred to a gynaecologist on account of this. On examination under anaest however that mass was found to be a full bladder.

The urinary infection had recurred and was again treated with sulphonam Retention of urine was troublesome and she was given carbachol for this. Intrave pyelography was normal. Cystoscopy showed trabeculation of the bladder wall thickening of the base. She was discharged and given an appointment to at Mr. Miller's Out-Patients on 31st October.

When she arrived at Out-Patients on 31st October she was desperately ill. She stuporose, just rousable but unable to answer any questions. Her limbs mo normally. Gross dehydration was present. She had pressure sores over her sacrum her heels. Respirations were regular and *not* of the acidotic variety. The blood-pres was 70/40, the pulse 70/min. and the extremities cold and dry. Her husband said had been in this condition for the past 24 hours and also that she had been unabl eat or drink for the past week. However, all this time she was receiving 18 uni P.Z.I. every morning from the district nurse.

The urine was found to contain thick pus, no sugar and a moderate quantit ketones. The blood sugar was found to be 740 mg. per cent. and the blood urea 540 per cent.

This then was a case of gross dehydration with hyperglycaemia, but little ket and a severe urinary infection and uraemia.

She was treated with insulin, some of which was given intravenously, streptom for the urinary infection and intravenous saline. It was decided to rehydrate rapidly and 3 litres of saline were given in 2 hours. After this the neck veins beg distend and the drip was slowed down. By noon the next day the blood sugar was mg. per cent. and at this time she had received a total of 300 units of insulin. The bl pressure however remained low and the haemoglobin was found to be only 75 per c One pint of blood was transfused but this had no effect on the blood-pressure.

PLATE I

*The right kidney is full of abscesses, some of which have almost penetrated the capsule. In both kid*[illegible]
*a pale wavy line marks the limit of each necrotic papilla.*

By the second day she was considered to be rehydrated. Her urine output however d been only 240 c.c. in 24 hours and the blood-pressure was still low. It was decided raise the blood-pressure by means of noradrenaline given in a slow intravenous fusion. Eight mg. of noradrenaline in a litre of saline were given. The blood-essure rose on this to 130/70 and this was maintained for the next 24 hours. At the d of this period pitting oedema was present over the sacrum and pubis and although e blood-pressure was maintained the general condition remained poor with the patient iporose but restless. The drip was therefore stopped and morphia given. When the ip was taken down it was noticed that there was a white line running up the phenous vein from the site of the noradrenaline drip.

*Professor C. B. Perry*: She had a blood-sugar 700 mg. per cent. and no reducing bstances in the urine, how do you account for this?

*Dr. McInnes*: It might have been due to the large numbers of organisms in the urine.

*Professor C. B. Perry*: You don't think it was due to the low blood-pressure?

*Dr. McInnes*: It may have been so.

*Dr. Cates*: Could you tell us more about the character of the abdominal pain in ugust?

*Dr. McInnes*: It was very severe, stabbing in character and radiating from the domen to the limbs. There was a tight feeling across the abdomen. The pain woke r in the middle of the night. There was no haematuria with the abdominal pain.

*Professor C. B. Perry*: Why was insulin given intravenously?

*Dr. McInnes*: Because it was thought that subcutaneous would not be absorbed as e was so shocked.

*Professor C. B. Perry*: I dont think intravenous insulin is any use as it is all excreted rapidly. Diabetic coma does not respond so well to intravenous as to subcutaneous sulin.

*Dr. Cates*: There are two schools of thought and it is probably advisable to give oth subcutaneous and intravenous insulin.

*Professor C. B. Perry*: If you are going to give it intravenously it is very important to ve it into the bottle and not into the tubing.

*Professor Hewer*: At post mortem the body appeared in a very sorry state. There was rly gangrene of all the toes and bedsores were present over the sacrum, heels, elbows d indeed over all the pressure points.

On the left leg, at the site of the noradrenaline drip, there was a white line extending the length of the leg; for 1.5 cm. on either side of this line was a plum-coloured ea. This tissue felt soft and inelastic to touch and entirely different from the adjacent in. Sections of this skin show it to be necrotic. The vessels do not contain blood but ly a mesh-work of fibrin and leucocytes. The skin itself has not sloughed as the hole process is quite recent. This shows the importance of giving noradrenaline to a moving blood-stream and not into an empty vein as occurs in a "Cut-down.

The lungs showed collapse of both lower lobes and there was frank abscess for-ation in the medial part of the right basal segments. Culture grew a coagulase sitive staphylococcus aureus. A staphylococcus was also grown from a thrombosed edian cubital vein in the right arm which had been used for an intravenous drip. his staphylococcus was of the same phage type as the one isolated from the lung.

The kidneys were the most interesting organs at post mortem. The capsules were ry thick and adherent. Both kidneys contained masses of confluent abscesses; the ght kidney was more severely affected than the left, however. This was an acute elonephritis and a coliform bacillus was isolated from the abscess. As this patient d both anuria and diabetes, renal papillary necrosis was suspected and the cut rface of the kidneys shows this to be present. Every one of the papillae is affected but ne of them has sloughed. There is a well-marked white line of leucocytic infiltration rrounding the necrotic areas which are infarcted. This process has included the pillae and about two thirds of the width of the medulla (Plate I).

There is some dilatation of both pelves and ureters which are intensely congested and inflamed. Sections of the kidney show multiple subcapsular abscesses and infar of the renal papillae. The renal tissues are full of pus. Clumps or colonies of ba are growing in the infarcted tissue.

The bladder was dilated and hypertrophied and as there was no obvious sig obstruction it was decided to examine the bladder neck with particular care. This dition is very difficult to assess at post mortem but histologically in this case t appears to have been an excess of muscle and I suggest that bladder neck obstru must have been present.

This then was a case of diabetes mellitus which was poorly controlled and w developed a severe urinary infection possibly complicating a bladder neck obstruc Diabetic coma and uraemia competed with each other in causing her death.

*Professor Neale*: There was no evidence of endocrine abnormality?

*Professor Hewer*: None.

*Professor Perry*: Perhaps the original urinary infection was never eradicated. does renal papillary necrosis occur?

*Professor Hewer*: The complete answer is not known. There is no special b supply and the vessels in the region of the necrosis show no specific ch they just end suddenly at the point of thrombosis. There is a muscle which surro and encircles the papillae in a spiral fashion and it is an attractive theory tha necrosis is caused by spasm of this muscle. Against this theory is the fact tha necrosis occurs much higher in the medulla than the muscle.

Dr. Cates and I are writing a paper ([1]) on this condition with a description of other cases that have come our way. Urinary infections are of course well recog as a hazard in diabetes and renal papillary necrosis sometimes supervenes and c a sudden aggravation of the condition. It is frequently an autopsy finding perhaps of no great consequence but sometimes recovery takes place and the whence the papilla sloughed heals and allows urine to escape from the colle tubules.

### REFERENCE

[1] *British Medical Journal*, 1956, i, pp. 1005-8

## Figures and Tables

**Figure f1:**